# Performance of ChatGPT-4o in Determining Radiology–Pathology Concordance and Management Recommendations Following Image-Guided Breast Biopsies

**DOI:** 10.3390/diagnostics15192536

**Published:** 2025-10-08

**Authors:** Albert Lee, Belinda Curpen, Afsaneh Alikhassi

**Affiliations:** 1 Temerty Faculty of Medicine, University of Toronto, Toronto, ON M5S 3K3, Canada; 2 Medical Imaging, Sunnybrook Health Science Centre, Toronto, ON M4N 3M5, Canada

**Keywords:** ChatGPT, radiology-pathology concordance, breast biopsy, artificial intelligence, diagnostic concordance, large language model

## Abstract

**Background:** Determining radiology–pathology concordance after breast biopsies is critical to ensuring appropriate patient management. However, expertise and multidisciplinary input are not universally accessible. **Purpose:** To evaluate the performance of a large language model, ChatGPT-4o, in determining the radiology–pathology concordance of breast biopsies and suggesting subsequent management steps. **Methods:** A retrospective single-center study analyzed 244 cases of image-guided breast biopsies of women. ChatGPT-4o assessed de-identified radiology and pathology reports for concordance and recommended management. Radiologist assessments served as the reference standard with final surgical pathology and 2-year imaging follow-up serving as gold standards when applicable. Concordance rates, management recommendations, and diagnostic agreement with the gold standard were compared using statistical tests, including McNemar’s, chi-square, Fisher–Freeman–Halton, and Cohen’s kappa. **Results:** ChatGPT-4o achieved a concordance rate of 98.8% vs. 98.0% for radiologists (*p* = 0.625) and demonstrated high diagnostic agreement with the gold standard (kappa = 0.947, *p* < 0.001). ChatGPT-4o favored imaging follow-up more than radiologists (49.2% vs. 41.8%, *p* < 0.001) and surgical management less frequently (41.8% vs. 46.7%). **Conclusions:** ChatGPT-4o demonstrated diagnostic performance comparable to radiologists with breast imaging subspecialities in evaluating breast biopsy concordance. Its slightly more conservative management approach may enhance shared decision-making in resource-limited settings.

## 1. Introduction

In current practice, the role of the radiologist extends beyond performing image-guided breast biopsies; it also includes assessing the concordance between radiology and pathology findings [1,2,3]. This evaluation is essential for determining the appropriate next steps in patient care. Errors in judging concordance can lead to missed diagnoses and unfavorable patient outcomes [1,2,3,4,5]. For instance, if a biopsy is performed on a suspicious group of calcifications, but the pathology results show only fibrocystic changes, an addendum report from the radiologist is critical. This report helps guide the clinician and the patient in deciding the next course of action, which may involve repeating the biopsy, possibly with a larger bore needle.

In many academic hospitals, there are dedicated breast centers with breast fellowship-trained radiologists, pathologists, and surgeons. However, there are many medical centers without this expertise or availability of a multidisciplinary board to discuss complex cases [6,7]. The potential for advanced artificial intelligence (AI) models, such as ChatGPT, to assist in these radiologic–pathologic results and recommend further management offers a promising avenue for enhancing clinical decision-making and improving patient care.

Recent advancements in AI present an opportunity to leverage large language models (LLMs) to assist in the interpretation of medical data and support clinical decision-making [8,9]. Several studies have explored the performance of ChatGPT and other LLMs in radiological decision-making [10,11,12], including assessing ChatGPT’s potential to provide useful recommendations for radiological imaging decisions based on textual information [10], describe mammographic findings, and assign BI-RADS categories based on imaging reports [13,14]. These studies use domain-specific terminologies and make decisions based on structured reports, laying the groundwork for ChatGPT’s potential in more complex tasks. Limited research has explored its ability to perform well on theoretical radiology tasks. This study investigates whether ChatGPT-4o can determine radiology–pathology concordance and provide appropriate management recommendations, potentially offering decision support in settings with limited expert access.

## 2. Materials and Methods

This retrospective single-center study was approved by the institutional ethics committee (Project ID 6382), where the coordinating center is located, and the need for specific informed consent was waived due to the use of fully anonymized data.

### 2.1. Study Design and Setting

This retrospective single-center study was conducted at Sunnybrook Health Sciences Centre, Toronto, ON, Canada, a tertiary referral hospital, reviewing breast biopsy cases between September 2022 and December 2022. The study included full-length descriptive radiology reports for women who underwent image-guided breast biopsies under ultrasound, stereotactic/tomosynthesis, or MRI. All breast imaging reports were produced by 5 board-certified fellowship-trained breast radiologists. Reports were written in English (the dominant language of the training data of ChatGPT-4o). Corresponding to the breast biopsy cases, full-length English pathology reports were also acquired, which were produced by board-certified dedicated breast pathologists.

**Figure 1:**
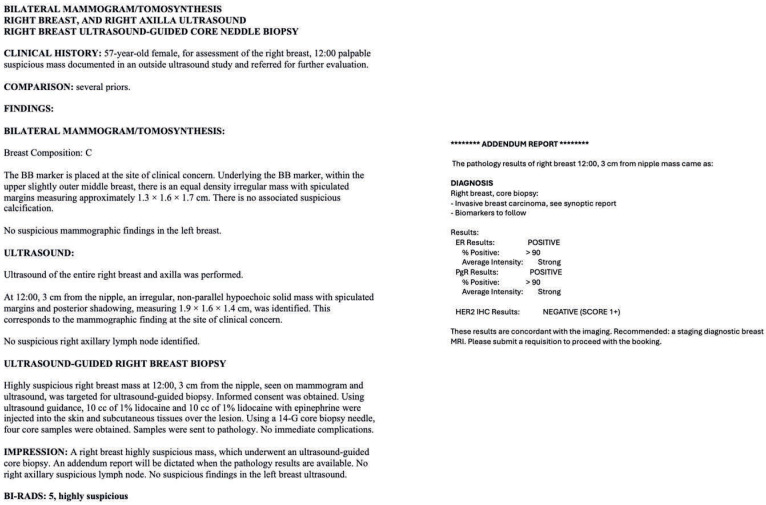
(**A**) An example of descriptive radiology reports, which were input into ChatGPT-4. (**B**) The radiologist’s addendum report for the same patient. The inclusion criteria were women who had undergone image-guided breast biopsies under ultrasound, stereotactic/tomosynthesis, or MRI with complete reports. Complete reports must include the radiology report, pathology report, radiology–pathology concordance, and radiologist’s management recommendations after radiologic–pathologic concordance. Patients with incomplete records, including those without a descriptive radiology report describing the pre-biopsy breast lesion, were excluded from the study. Male cases were also excluded to prevent re-identification, given their low prevalence. Other exclusion criteria include non-breast biopsies performed, such as axillary lymph node biopsies/fine-needle aspirations and cases that were lost to two-year follow-up. Additionally, cases that were biopsied benign but had concurrent ipsilateral breast cancer and underwent mastectomy were also excluded from the study, because it was not possible to correlate the biopsied lesions with the final surgical pathology on mastectomy (Figure 2). There was no case with the procedural report mentioning inadequate biopsy sampling or inappropriate positioning of the biopsy clip.

The descriptive radiology reports, which followed BI-RADS guidelines, were input into ChatGPT-4o and included patient demographics, clinical context, the imaging findings of the breast, BI-RADS classification, and the modality under which the biopsy was performed (Figure 1). For instance, we provided a descriptive MRI radiology report for a breast mass that underwent an MRI-guided biopsy. The report included details about the patient’s age and clinical context, such as the detection of a new enhancing mass during a high-risk screening MRI. Additionally, we described the lesion’s characteristics, including its shape, margins, enhancement, kinetic features, and size. The radiologists’ assessment of radiology–pathology concordance and recommended next management steps were also recorded.

Radiology–pathology: Definition of discordance: Discordance between imaging and histopathological reports occurs when the histological results do not adequately explain the features observed in the imaging studies. Discordant lesions include instances where the findings strongly suggest malignancy but ultimately yield benign results.

### 2.2. Report-Processing and Data Collection

Descriptive radiology reports and breast biopsy pathology reports were exported into Excel spreadsheets 2024S (Microsoft). A single reader (A.L.) reviewed reports to remove patient-identifying information. The full-length original text, including any topographical errors, was left unaltered. The BI-RADS category and radiologists’ assessment of radiology–pathology concordance and next management steps were recorded (5 radiologists, ranging from 5 to 28 years of experience). Pathology reports of the breast biopsies were categorized into benign, high-risk, and malignant. Breast high-risk lesions identified in core biopsies are abnormalities in breast tissue that are linked to a higher long-term risk of developing breast cancer in either breast and/or an increased risk of upgrading to invasive cancer or carcinoma in situ when surgically excised. Therefore, we categorized cases of atypical ductal hyperplasia (ADH) and atypical lobular hyperplasia (ALH) within this group. We did not have radial scars, papillomas with atypia, or lobular carcinomas in situ. Benign papillomas without atypia were considered benign and ductal carcinomas in situ (DCIS) were categorized as malignant.

To set a gold standard, final surgical pathologies were also collected when applicable for the appropriately biopsied breast lesions. If the lesion biopsied did not undergo surgical excision, then a 2-year imaging follow-up was set as the gold-standard diagnosis. If a lesion was resolved or unchanged after 2-year imaging follow-up, it would be categorized as the same as when it was initially biopsied (e.g., benign lesions would remain benign and high-risk lesions would remain high-risk).

### 2.3. ChatGPT-4o Integration

Publicly available ChatGPT-4o was used as the large language model to assess the objectives of this study. No training was performed on ChatGPT-4o. The model assessed manually input de-identified matched full-length radiology and pathology reports and determined concordance and suggested management steps. The following prompts were input before the radiology and pathology reports: “Given the following radiology and pathology reports, is there radiology–pathology concordance? What is the single best next management step?”. No fine-tuning was performed. This process was performed between 26 February and 24 April 2025. ChatGPT-4o was blinded to radiologist-determined concordance and management decisions. There were no listed management options prompted for ChatGPT-4o. A new chat window would be refreshed after recording each sample to limit the possibility of learning and bias. Furthermore, there was no evidence of any difference in the performance of ChatGPT-4o when the same cases were presented to it again after a week.

### 2.4. Statistical Analysis

Descriptive statistics were used to summarize the characteristics of the study cohort. For inferential statistics, comparisons between radiologist and ChatGPT-4o assessments were conducted using appropriate tests based on the nature of the data. McNemar’s test was used to compare paired categorical outcomes between the radiologist and ChatGPT-4o in concordance assessments. The Pearson Chi-Square test was employed to evaluate differences in management recommendations. Additionally, the Fisher–Freeman–Halton exact test was applied to assess biopsy pathology diagnosis agreement against the gold standard. To quantify the level of agreement beyond chance between ChatGPT-4o and the gold standard, the Kappa test was performed. This test used pathology assigned based on ChatGPT-4o’s recommended management, with more passive managements (imaging follow-up and clinical follow-up) assigned as benign and management requiring further actionable steps (staging/diagnostic MRI, re-biopsy, and surgical consult) assigned as high-risk or malignant, based on biopsy pathology.

A *p* value of less than 0.05 was considered statistically significant. All statistical analyses were performed using IBM SPSS Statistics for Windows, version 27.0.1 (IBM Corp., Armonk, NY, USA).

## 3. Results

### 3.1. Summary of Patient Demographics, Radiologist and ChatGPT-4o Assessments, Management Recommendations, and Diagnostic Concordance

The study included a total of 244 patients with a mean age of 57.05 years. The most used imaging modality for biopsy was ultrasound (156 cases, 63.9%), followed by MRI (45 cases, 18.4%) and stereotactic/tomosynthesis imaging (43 cases, 17.6%). The majority of the biopsies were categorized as BI-RADS 4 (80.7%), with fewer cases categorized as BI-RADS 5 (19.2%). ChatGPT-4o assessed the pathology results as concordant in 98.8% of cases, compared with 98.0% for the radiologists. Radiologists most frequently recommended surgical management/consult (46.7%) and imaging follow-up (41.8%), while ChatGPT-4o suggested imaging follow-up more often (49.2%) and surgical management/consult slightly less frequently (41.8%). Regarding final outcomes, breast biopsy pathology diagnosis was benign in 56.6% of cases, high risk in 6.6%, and malignant (cancer/DCIS) in 36.9%. The gold-standard diagnosis was nearly identical to the biopsy pathology with benign in 56.6% of cases, high risk in 4.5%, and malignant in 38.9% (Table 1).

### 3.2. Comparison of Concordance Assessments Between Radiologists and ChatGPT

Concordance was high for both radiologists and ChatGPT-4o, with radiologists determining a concordance rate of 98.0% and ChatGPT-4o slightly higher at 98.8%. Discordant cases were rare, occurring in only 2.0% of radiologist assessments (*N* = 5) and 1.2% of ChatGPT-4o assessments (*N* = 3). The difference in concordance between the two was not statistically significant (*p* = 0.625), indicating comparable performance in diagnostic agreement. These findings suggest that ChatGPT-4o may serve as a reliable tool in assessing radiology–pathology concordance, with performance closely matching that of fellowship-trained breast radiologists (Table 2).

There were 4 cases out of 244 that had differences between the radiologists’ and ChatGPT-4o’s assessments for radiology–pathology concordance. Of all the cases, 1 out of 239 radiologist-assessed concordant cases were deemed discordant by ChatGPT-4o, and 3 of the 5 radiologist-assessed discordant cases were deemed concordant by ChatGPT-4o (Supplementary Table S1). Further analyses of these specific cases are described later in the results section.

### 3.3. Comparison of Radiologist and ChatGPT-4o Management Recommendations

Radiologist and ChatGPT-4o management recommendations showed notable differences across several categories. Both suggested clinical follow-up in 1.2% of cases, while ChatGPT-4o recommended imaging follow-up more frequently (49.2%) compared with radiologists (41.8%). Conversely, radiologists were more likely to recommend surgical intervention (46.7%) than ChatGPT-4o (41.8%). MRI staging or diagnostic use was also slightly higher among radiologists (8.6%) than ChatGPT-4o (6.6%). The difference in the overall distribution of management recommendations was statistically significant (*p* < 0.001). These findings suggest that while ChatGPT-4o closely aligns with expert judgment in some categories, it tends to favor less invasive management strategies and may represent a more conservative decision-making approach in certain clinical scenarios (Table 3).

**Figure 3:**
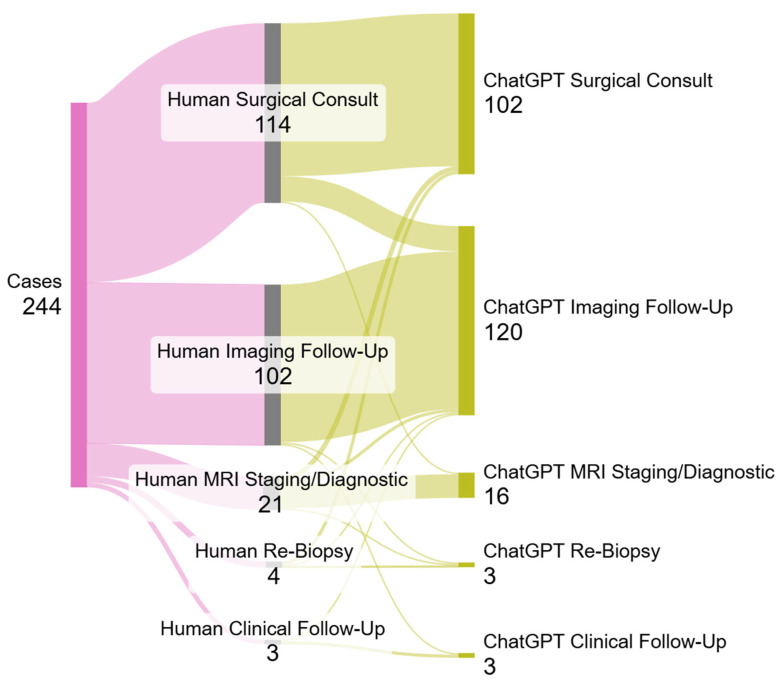
Sankey plots showing changes in management recommendations between human readers (radiologists) and ChatGPT-4o. The difference in overall distribution of management recommendations was statistically significant (*p* < 0.001).

The largest discrepancy between radiologists’ and ChatGPT-4o’s recommendations for management were cases where the radiologist recommended surgical management/consult and ChatGPT-4o recommended imaging follow-up (*N* = 16). This was followed by cases where the radiologist recommended staging/diagnostic MRI and ChatGPT-4o recommended surgical consult (*N* = 3) (Figure 3, Supplementary Table S1).

### 3.4. Comparison of Biopsied Pathology Diagnoses Against Gold-Standard Diagnoses

Comparison of breast biopsy pathology diagnoses with the gold standard revealed a high diagnostic alignment. Among cases classified as benign by biopsy, all 138 were correctly identified as benign after 2 years of imaging follow-up or surgical pathology. For high-risk lesion cases, 11 (68.8%) remained high-risk after 2 years of imaging follow-up or surgical pathology, with 5 (31.2%) cases upgraded to malignant. All 90 malignant cases were correctly identified on final surgical pathology. These distributions were statistically significant (*p* < 0.001) (Table 4).

### 3.5. Kappa Statistics for Agreement ChatGPT-4o Diagnoses with Gold-Standard Diagnoses

The agreement between ChatGPT-4o’s presumed diagnosis based on management with the gold-standard diagnosis was assessed using Kappa statistics. ChatGPT showed an excellent level of agreement, with Kappa values of 0.947 and an approximate T-value of 17.312 (SE = 0.020), indicating almost perfect concordance. This agreement was statistically significant (*p* < 0.001), reflecting a high consistency between diagnostic assessments and the gold standard (Table 5).

### 3.6. Special Cases of Differences Between Radiologists and ChatGPT-4o

Of the cases, 1 out of 239 radiologist-assessed concordant cases were determined to be discordant by ChatGPT-4o. This was a case of MRI-guided biopsy where the pathology yielded breast-parenchyma. For this case, the radiologist recommended imaging follow-up and ChatGPT-4o recommended re-biopsy. The lesion was benign by the gold standard (stable after 2-year imaging follow-up).

Three of the five radiologist-assessed discordant cases were determined to be concordant by ChatGPT-4o. Two of the three cases were masses biopsied under ultrasound-guidance and one case was of calcifications under stereotactic guidance. All biopsied pathologies yielded benign results, with the radiologists recommending surgical consult in two cases and re-biopsy in one case. The re-biopsied case yielded the same pathology, and all three cases were followed with imaging for 2 years. ChatGPT-4o’s management recommendations for all these cases were imaging follow-up. The lesions were all benign by the gold standard.

There were 16 cases where the radiologist recommended surgical management/consult and ChatGPT recommended imaging follow-up. Of these 16 cases, 2 had differing concordance–discordance assessments between radiologists and ChatGPT-4o, which were accounted for above (radiologist discordance vs. ChatGPT-4o concordance). The remaining 14 cases were radiology–pathology concordant, which were agreed upon by both radiologists and ChatGPT-4o. In total, 9 of the 14 cases were biopsied as papilloma, and 5 of the 14 cases were biopsied as fibroadenomas. The gold standard for all 14 cases confirmed benign pathology.

We had 16 high-risk lesion biopsies yield ALH or ADH. Five high-risk lesions upgraded to invasive breast carcinoma in surgical pathology. All five cases were assessed to be radiology–pathology concordant by both radiologists and ChatGPT-4o. In four out of the five cases, surgical management/consult was recommended by both radiologists and ChatGPT-4o. There was one single case of ALH in which the radiologist recommended MRI, while ChatGPT-4o suggested surgical consultation.

## 4. Discussion

This study demonstrates that ChatGPT-4o performs comparably to fellowship-trained breast radiologists in determining radiology–pathology concordance following image-guided breast biopsies. The model achieved a concordance rate of 98.8%, statistically equivalent to the radiologists’ rate of 98.0% (*p* = 0.625) and showed excellent agreement with the gold-standard diagnosis (κ = 0.947). These findings suggest that ChatGPT has the potential to serve as a valuable adjunct in radiology–pathology correlation.

Interestingly, although concordance performance was similar, ChatGPT-4o demonstrated a statistically significant difference in management recommendations (*p* < 0.001), favoring imaging follow-up more frequently than radiologists (49.2% vs. 41.8%) and recommending surgical management less often (41.8% vs. 46.7%). This trend reflects a more conservative approach by the model and may be influenced by risk-averse tendencies embedded in its training data. Notably, in 14 radiology–pathology concordant cases where ChatGPT-4o recommended imaging follow-up and the radiologist advised surgical consultation, all cases were ultimately benign (primarily papillomas and fibroadenomas). While conservative follow-up may be clinically appropriate in many such cases, overt reliance on algorithmic recommendations could delay intervention where clinical context or imaging–pathologic discordance warrants escalation.

The few discordant cases between radiologist and ChatGPT-4o assessments underscore the nuanced reasoning that underlies concordance decisions. For example, ChatGPT-4o identified one case as discordant that the radiologist deemed concordant; the final benign outcome after 2-year follow-up suggests ChatGPT-4o’s suggestion for re-biopsy may have been overly cautious. Conversely, ChatGPT-4o interpreted three radiologist-labeled discordant cases as concordant—all of which were benign at follow-up—suggesting that in select scenarios, ChatGPT-4o may appropriately avoid unnecessary intervention. These examples highlight the promise and pitfalls of LLM-based tools in complex diagnostic workflows and reinforce the importance of human oversight.

These findings build on prior research demonstrating ChatGPT’s capacity to perform radiologic reasoning and triage [10,11,12,13], now extending its utility into high-stakes diagnostic reconciliation tasks. This study is among the first to empirically validate ChatGPT’s role in radiology–pathology concordance—a domain requiring integration of clinical, imaging, and histopathologic data.

Several limitations exist in this study. First, ChatGPT-4o assessments were based on de-identified text inputs and lacked clinical metadata, patient history, or imaging visuals, all of which may influence expert decisions. Although this isolation strengthens the model’s reproducibility, it limits ChatGPT’s ability to contextualize findings as a radiologist might. Second, while ChatGPT-4o was not fine-tuned or trained on these data, inherent biases in its general training corpus may influence management tendencies. Third, this single-center study reflects practice patterns at a tertiary academic institution, and the findings may not generalize to community settings or different LLM versions. Fourth, the sample size was relatively small with a low number of radiologist- and ChatGPT-assigned discordant cases.

Future research should explore multimodal models capable of incorporating imaging, patient history, and pathology in real time, as well as integration within clinical workflows through human–AI collaboration frameworks. Prospective validation in real-world settings, cost–benefit analyses, and ethical considerations—including transparency, accountability, and explainability—must guide responsible AI implementation.

## 5. Conclusions

ChatGPT-4o demonstrated a diagnostic performance equivalent to breast radiologists in assessing radiology–pathology concordance following image-guided breast biopsies. While its slightly more conservative management recommendations diverged from radiologists’ choices, these differences largely reflected variation in benign cases and did not compromise diagnostic accuracy. These findings suggest that large language models such as ChatGPT may offer reliable support for concordance assessment and management planning—particularly in settings with limited access to subspecialized expertise. With appropriate oversight and integration, LLMs may serve as valuable tools in augmenting breast-imaging workflows, supporting equitable, high-quality care across diverse practice environments.

## Figures and Tables

**Figure 2:**
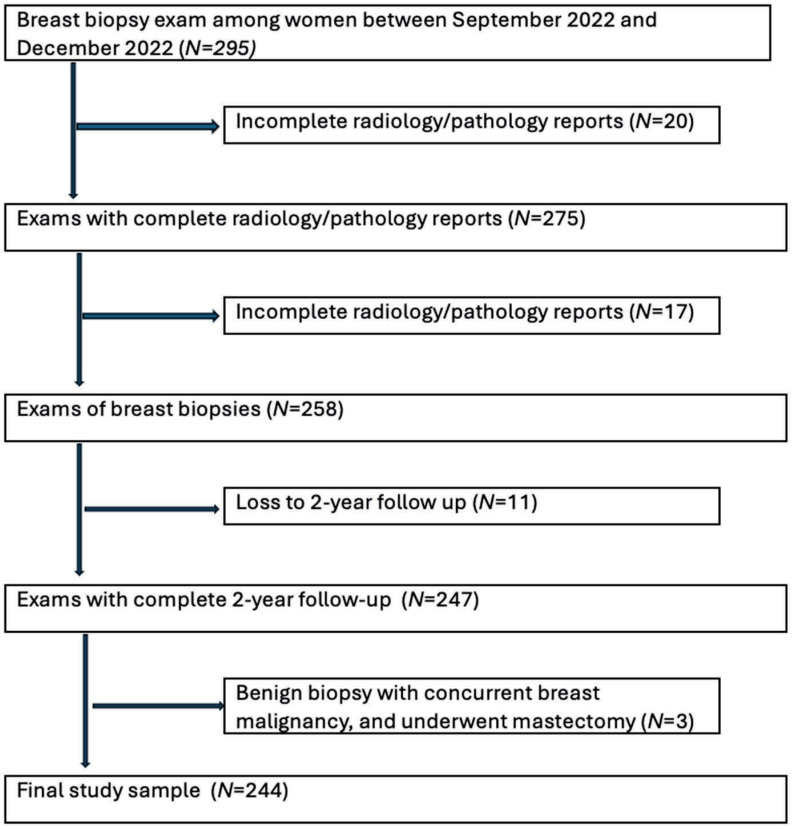
Inclusion and exclusion flowchart.

**Table 1:** Summary of patient demographics, radiologist and ChatGPT assessments, management recommendations, and diagnostic concordance. The numbers in parentheses are percentages.

		Mean	SD	*N*
Patient Age (Years)		57.05	14.85	
Cases Per Radiologist		48.8	1.92	
Imaging Modality	Ultrasound			156 (63.9)
	MRI			45 (18.4)
	Stereotactic/Tomosynthesis			43 (17.6)
BI-RADS Score (Radiologist’s Assessment Category)	4			188 (77.0)
	5			56 (23.0)
**Radiologist’s Assessment**				
Radiologist’s Concordance Assessment	Discordant			5 (2.0)
	Concordant			239 (98.0)
Radiologist Management Recommendation	Surgical Consult			114 (46.7)
	Imaging Follow-up			102 (41.8)
	MRI Staging/Diagnostic			21 (8.6)
	Re-biopsy			4 (1.6)
	Clinical Follow-up			3 (1.2)
**ChatGPT’s Assessment**				
ChatGPT’s Concordance Assessment	Discordant			3 (1.2)
	Concordant			241 (98.8)
ChatGPT’s Management Recommendation	Surgical Consult			102 (41.8)
	Imaging Follow-up			120 (49.2)
	MRI Staging/Diagnostic			16 (6.6)
	Re-biopsy			3 (1.2)
	Clinical Follow-up			3 (1.2)
**Cancer Status**				
Biopsy Pathology Diagnosis	Benign			138 (56.6)
	High Risk			16 (6.6)
	Malignant			90 (36.9)
Gold-standard Diagnosis	Benign			138 (56.6)
	High Risk			11 (4.5)
	Malignant			95 (38.9)

**Table 2:** Comparison of concordance assessments between radiologists and ChatGPT.

	Radiologist’s Concordance Assessment	ChatGPT’s Concordance Assessment	*p* Value ^M^ *
Discordant	5 (2.0)	3 (1.2)	0.625
Concordant	239 (98.0)	241 (98.8)	

^M^ McNemar’s Test, * *p* < 0.05, Significant.

**Table 3:** Comparison of radiologist and ChatGPT management recommendations. The numbers in parentheses are percentages.

	Radiologist Management Recommendation	ChatGPT’s Management Recommendation	*p* Value ^C^ *
Surgical Consult	114 (46.7)	102 (41.8)	<0.001
Imaging Follow-up	102 (41.8)	120 (49.2)	
MRI Staging/Diagnostic	21 (8.6)	16 (6.6)	
Re-biopsy	4 (1.6)	3 (1.2)	
Clinical Follow-up	3 (1.2)	3 (1.2)	

^C^ Pearson Chi-Square test, * *p* < 0.05, Significant.

**Table 4:** Comparison of biopsy pathology diagnoses against gold standard diagnosis.

		Gold-Standard Diagnosis						
		Benign		High-Risk		Malignant		*p* Value ^F^ *
		*N*	Column %	*N*	Column %	*N*	Column %	
Biopsy Pathology Diagnosis	Benign	138	100.0%	0	0.0%	0	0.0%	<0.001
	High Risk	0	0.0%	11	100.0%	5	5.3%	
	Malignant	0	0.0%	0	0.0%	90	94.7%	

^F^ Fisher–Freeman–Halton Exact test; * *p* < 0.05, Significant.

**Table 5:** Kappa statistics for agreement of ChatGPT recommendations, presuming diagnosis, with gold-standard diagnosis.

Symmetric Measures					
		Value	Asymptotic Standard Error ^a^	Approximate T ^b^	*p* Value *
ChatGPT	Kappa	0.947	0.020	17.312	<0.001
	*N*	244			

^a^ Not assuming the null hypothesis. ^b^ Using the asymptotic standard error assuming the null hypothesis. * p < 0.05, Significant.

## Data Availability

The data presented in this study are available on request from the corresponding author, due to our institute’s policy and protecting patients’ health information.

## Supplementary Materials

The following supporting information can be downloaded at https://www.mdpi.com/article/10.3390/diagnostics15192536/s1, Table S1. Differences of concordance assessment and management recommendations between radiologists and ChatGPT; Table S2. Recommendations of Radiologists vs ChatGPT for BIRADS 4 lesions with percentages calculated based on total BI-RADS 4 lesions; Table S3. Recommendations of Radiologists vs ChatGPT for BIRADS 5 lesions with percentages calculated based on total BI-RADS 5 lesions.
