# Supplementary material for: Performance of ChatGPT-4o in Determining Radiology–Pathology Concordance and Management Recommendations Following Image-Guided Breast Biopsies
Source: Diagnostics (Basel). 2025 Oct 8;15(19):2536. doi: 10.3390/diagnostics15192536 (PMC12523907; doi:10.3390/diagnostics15192536)
Supplement: Supplementary file 1 [file diagnostics-15-02536-s001.zip › diagnostics-3899550-supplementary.pdf]

Supplemental Table S1. Differences of concordance assessment and management recommendations between radiologists and ChatGPT. The numbers in parentheses are percentages.

Difference Between Radiologist and ChatGPT		
Difference in Concordance Assessment	Same	240 (98.4)
	Different	4 (1.6)
	Human Concordant to ChatGPT Discordant	1 (0.4)
	Human Discordant to ChatGPT Concordant	3 (1.2)
Difference in Management Recommendation	Same	215 (88.1)
	Different	29 (11.9)
	Human Surgical Consult to ChatGPT Imaging Follow-Up	16 (6.6)
	Human Surgical Consult to ChatGPT MRI Staging/Diagnostic	1 (0.4)
	Human Imaging Follow-Up to ChatGPT Re-biopsy	1 (0.4)
	Human Imaging Follow-Up to ChatGPT Clinical Follow-Up	1 (0.4)
	Human MRI Staging/Diagnostic to ChatGPT Surgical Consult	3 (1.2)
	Human MRI Staging/Diagnostic to ChatGPT Imaging Follow-Up	2 (0.8)
	Human MRI Staging/Diagnostic to ChatGPT Re-Biopsy	1 (0.4)
	Human Re-Biopsy to ChatGPT Surgical Consult	2 (0.8)
	Human Re-Biopsy to ChatGPT Imaging Follow-Up	1 (0.4)
	Human Clinical Follow-Up to ChatGPT Imaging Follow-Up	1 (0.4)

Supplemental Table S2. Recommendations of Radiologists vs ChatGPT for BI-RADS 4 lesions with percentages calculated based on total BI-RADS 4 lesions.

BI-RADS 4 (N = 197)			
		**Radiologist**	**ChatGPT**
Benign (N = 138)	Surgical Consult	25 (12.7)	12 (6.1)
	Imaging Follow Up	101 (51.3)	117 (59.4)
	MRI Staging/Diagnostic	7 (3.6)	4 (2.0)
	Re-biopsy	2 (1.0)	2 (1.0)
	Clinical Follow-Up	3 (1.5)	3 (1.5)

High-Risk (N = 16)	Surgical Consult	11 (5.6)	12 (6.1)
	Imaging Follow Up	1 (0.5)	3 (1.5)
	MRI Staging/Diagnostic	2 (1.0)	0
	Re-biopsy	2 (1.0)	1 (0.5)
	Clinical Follow-Up	0	0
Malignant (N = 43)	Surgical Consult	40 (20.3)	40 (20.3)
	Imaging Follow Up	0	0
	MRI Staging/Diagnostic	3 (1.5)	3 (1.5)
	Re-biopsy	0	0
	Clinical Follow-Up	0	0

Supplemental Table S3. Recommendations of Radiologists vs ChatGPT for BI-RADS 5 lesions with percentages calculated based on total BI-RADS 5 lesions. The numbers in parentheses are percentages.

**BI-RADS 5 (N = 47)**			
		**Radiologist**	**ChatGPT**
Benign (N = 0)	Surgical Consult	0	0
	Imaging Follow Up	0	0
	MRI Staging/Diagnostic	0	0
	Re-biopsy	0	0
	Clinical Follow-Up	0	0
High-Risk (N = 0)	Surgical Consult	0	0
	Imaging Follow Up	0	0
	MRI Staging/Diagnostic	0	0
	Re-biopsy	0	0
	Clinical Follow-Up	0	0
Malignant (N = 47)	Surgical Consult	38 (80.9)	38 (80.9)
	Imaging Follow Up	0	0
	MRI Staging/Diagnostic	9 (19.1)	9 (19.1)
	Re-biopsy	0	0
	Clinical Follow-Up	0	0
